# Device Free Detection in Impulse Radio Ultrawide Bandwidth Systems

**DOI:** 10.3390/s21093255

**Published:** 2021-05-08

**Authors:** Waqas Bin Abbas, Fuhu Che, Qasim Zeeshan Ahmed, Fahd Ahmed Khan, Temitope Alade

**Affiliations:** 1 Department of Electrical Engineering, National University of Computer and Emerging Sciences, Islamabad 44000, Pakistan; waqas.abbas@nu.edu.pk; 2 School of Computing and Engineering, University of Huddersfield, Huddersfield HD1 3DH, UK; fuhu.che@hud.ac.uk; 3 School of Electrical Engineering and Computer Science, National University of Sciences and Technology, Islamabad 44000, Pakistan; fahd.ahmed@seecs.edu.pk; 4 Computer Science at the Department of Computing and Technology, Nottingham Trent University, Nottingham NG11 8NS, UK; temitope.alade@ntu.ac.uk

**Keywords:** ultrawide bandwidth systems, Neyman–Pearson, probability of detection, probability of false alarm, characteristic function, signal processing

## Abstract

In this paper, an analytical framework is presented for device detection in an impulse radio (IR) ultra-wide bandwidth (UWB) system and its performance analysis is carried out. The Neyman–Pearson (NP) criteria is employed for this device-free detection. Different from the frequency-based approaches, the proposed detection method utilizes time domain concepts. The characteristic function (CF) is utilized to measure the moments of the presence and absence of the device. Furthermore, this method is easily extendable to existing device-free and device-based techniques. This method can also be applied to different pulse-based UWB systems which use different modulation schemes compared to IR-UWB. In addition, the proposed method does not require training to measure or calibrate the system operating parameters. From the simulation results, it is observed that an optimal threshold can be chosen to improve the ROC for UWB system. It is shown that the probability of false alarm, $P_{FA}$, has an inverse relationship with the detection threshold and frame length. Particularly, to maintain $P_{FA}<10^{-5}$ for a frame length of 300 ns, it is required that the threshold should be greater than 2.2. It is also shown that for a fix $P_{FA}$, the probability of detection $P_{D}$ increases with an increase in interference-to-noise ratio (INR). Furthermore, $P_{D}$ approaches 1 for INR >−2 dB even for a very low $P_{FA}$ i.e., $P_{FA}=1\times 10^{-7}$. It is also shown that a 2 times increase in the interference energy results in a 3 dB improvement in INR for a fixed $P_{FA}=0.1$ and $P_{D}=0.5$. Finally, the derived performance expressions are corroborated through simulation.

## 1. Introduction

COVID-19 has had a significant impact on our lifestyle [1]. Physical distancing, wearing masks, avoiding crowds, and maintaining better hygiene are becoming the new norms [2,3]. It has been advised to maintain at least a 1-meter distance between yourself and others [3]. Despite this, there are no concrete studies on the recommended distance between individuals in the indoor environment, where an intelligent guess would be more than 1 meter. However, at this moment, there are no particular devices that can help or provide accurate information of this distance to the user [4]. Furthermore, measurement of this distance becomes problematic, especially for indoor environments [4,5]. For indoor environments, when trying to measure this distance, two different approaches have been proposed: wearable [6] and device-free [7]. The wearable one is the most widely used approach, and the users are tracked by the sensor placed on the device [8,9,10]. The device-free approach has the ability to track people passively without having any contact with the person, resulting in a device-free communication [11,12,13]. Furthermore, device-free communications can provide more flexibility, comfort, and mobility compared to the wearable approach [14,15].

It is well known that the global positioning system (GPS) is a promising technology to provide outdoor positioning [16]. However, its limited penetration though solid objects makes it unreliable and inaccurate for indoor positioning [17]. To address this, several techniques including Wireless Fidelity (WiFi), Radio Frequency Identification(RFID), Bluetooth Low Energy (BLE) and ZigBee, where the localization is performed based on the received signal strength have been explored [18,19,20,21,22]. However, these techniques have drawbacks such as low accuracy, low data-rate and/or short range communication [18,19,20,21,22]. Furthermore, when using narrow-band device-free indoor communication, multipath fading and a cluttered environment make it difficult to design a low-cost and low-complexity receiver [23,24,25].

Ultrawide bandwidth (UWB) is a revolutionary low-power communication technology [26,27]. A UWB system offers many advantages such as high data rate, fading robustness, high precision ranging, obstacle penetration capability, and low-cost transceiver implementation [27,28,29,30,31]. All these advantages have made UWB a promising technique for device-free communications [24,25,26,32]. Initially, a UWB system was implemented with the help of a slow time hopping (STH) pulse position modulation (PPM) also known as the impulse radio (IR) [33]. However, different modulation schemes such as on–off keying (OOK) [34], pulse amplitude modulation (PAM) [34] and pulse shape modulation (PSM) [35,36], and different multiple access schemes such as direct-sequence (DS) [37] and hybrid Direct-Sequence and Time-Hopping (DS-TH) [38] have been implemented for UWB systems. In contrast to the received signal strength based positioning, UWB exploits either the time of arrival (ToA) or time difference of arrival (TDoA) for real-time positioning [39,40]. In [41], authors proposed an indoor positioning system based on UWB and long-range (LoRa) wireless technologies. Simulation results showed that the proposed system can achieve an accuracy of 15 cm. However, the proposed system was not investigated experimentally. The respiratory movement of a person is detected in laboratory conditions by using UWB radar, in [42]. The influence of a user state and environment was carried out in [43]. The performance of a UWB-based system by deploying anchor nodes on the floor was experimentally investigated in [44]. An elderly people tracking system based on the combination of UWB and Bluetooth low energy has been proposed in [45]. Artificial intelligence and machine learning techniques were developed in [39,40] to improve the indoor localisation to less than 10 cm by classifying them into line of sight (LoS) and non-LoS environments. All of these approaches used experimental demonstrations and no analytical framework was developed for a UWB system. An analytical framework was developed for UWB radar in [46]. However, this approach utilized the frequency domain technique to measure the vital signs such as breathing rate and heartbeat frequency. Cramer-Rao lower bounds (CRLBs) for estimation of signal parameters for single- and multi-path channel conditions were studied in [47].

In this paper, we develop a novel device-free indoor detection method that can be applied to IR-UWB networks. UWB technology is capable of localizing within 10–30 cm depending on different environments, where the indoor industrial environment is the most challenging due to a large number of reflections. By developing this UWB analytical framework, the device detection can be achieved with high accuracy followed by distance prediction, especially when operating in an LoS indoor environment. The development of such a framework at this moment is critical and will further lead to an accurate indoor localization system that is not present at the moment. This new method can be easily extended to existing device-free and device-based techniques and is easily applicable to any pulse-based UWB system using arbitrary multiple access or a modulation scheme. The main contributions of this paper are summarised as follows:

- The proposed detection method utilizes time domain concepts, which are different from the frequency-based approaches discussed in [39,40,42,43,46].
- In the context of using an UWB system as an indoor radar, the Neyman–Pearson (NP) criteria is applied. Detection probability and miss probability for a slow time hopping pulse position modulation (STH-PPM) system is developed.
- Characteristic function (CF) is utilized to measure the moments of the presence of the user. With the help of CF, higher orders of the moments can be calculated if required.
- No training is required to measure or calibrate the system operating parameters as needed in [24,25].

The rest of the paper is organized as follows. Section 2 describes the system model of the STH-PPM UWB system. The channel model, transmitted, and received signal is also discussed in this section. Section 3 help design the basic hypotheses testing for the proposed STH-PPM UWB system. In Section 4, the presence of the user is modelled and CF is utilized to measure the mean and variance of the user. The optimal threshold is also calculated in this section. In Section 5, the corresponding $P_{FA}$ and $P_{D}$ are derived. Performance analysis is carried out in Section 6. Finally, the paper is concluded in Section 7.

## 2. System Model

### 2.1. Transmitted Signal

Generally, a transmitted UWB signal is composed of $N_{s}$ pulses and is mathematically expressed as

(1) $$s\left(t\right)=\sum_{j=0}^{N_{s}-1}\psi \left(t-jT_{f}\right),$$

where ψ(·) denotes the UWB pulse, $T_{f}$ is the frame duration and *j* represents the frame index (the system is assumed to be a slow time-hopping system in which the number of frames and pulses is equal). Various different pulses, ψ(t), have been recommended for use in UWB systems. These pulses are derived based on a Gaussian pulse and its derivatives, modified hermite polynomial, and Gaussian modulated sinusoidal pulses, etc., and references within them [48,49].

### 2.2. Channel Model

The Saleh–Valenzuela (SV) channel model has been proposed for UWB communication in the 3 GHz to 10 GHz range [31,50,51,52,53,54]. The impulse response of the SV channel model is represented as

(2) $$\begin{array}{lll} \;h(t) & = & \sum_{v=0}^{V-1}\sum_{u=0}^{U-1}h_{u,v}\delta \left(t-T_{v}-T_{u,v}\right),\\ & = & \sum_{l=0}^{L-1}h_{l}\delta \left(t-T_{l}\right), \end{array}$$

where $h_{u,v}$ is the tap weight of the *u*-th ray in the *v*-th cluster, $T_{v}$ represents the delay of the *v*-th cluster, $T_{u,v}$ is the delay of *u*-th ray in the *v*-th cluster, *U* is the total number of rays and *V* is the total number of clusters experienced by the transmitted signal. As shown in (2), the rays of all the clusters can be expressed as *L* multipaths, where L=UV and $T_{l}$ represent the delay of the *l*-th multipath [55,56].

### 2.3. Received Signal

The received signal is expressed as

(3) $$\begin{array}{c} \;y\left(t\right)=\sum_{k=0}^{K-1}\sum_{l=0}^{L-1}\sum_{j=0}^{N_{s}-1}h_{l}\psi \left(t-jT_{f}-lT_{l}\right)+n\left(t\right), \end{array}$$

where the index *k* represents the *k*-th device. The noise n(t) is modelled as an additive white Gaussian noise with mean 0 and variance $\sigma^{2}$. Finally, the received signal of an STH-PPM system can be expressed as [26]

(4) $$\begin{array}{c} y=s+i+n, \end{array}$$

where

(5) $$\begin{array}{ccc} s & = & {\left[s_{\left(0,0\right)},s_{\left(0,1\right)},\cdots ,s_{\left(N_{s}-1,L-1\right)}\right]}^{T}, \end{array}$$

(6) $$\begin{array}{ccc} i & = & {\left[i_{\left(0,0\right)},i_{\left(0,1\right)},\cdots ,i_{\left(N_{s}-1,L-1\right)}\right]}^{T}, \end{array}$$

(7) $$\begin{array}{ccc} n & = & {\left[n_{\left(0,0\right)},n_{\left(0,1\right)},\cdots ,n_{\left(N_{s}-1,L-1\right)}\right]}^{T}, \end{array}$$

respectively. s is the desired signal, i denotes the effect of the present devices, n is the additive white Gaussian noise (AWGN) added at the receiver, $N_{s}$ denotes the repetition code length, *L* is the number of multipaths present in the system. (j,l) represents the signal of the *j*-th frame and *l*-th multipath. Presence or absence of a device can be determined by the following expression

(8) $$i_{\left(j,l\right)}=\left\{\begin{array}{cc} 0, & \;\mathrm{absence}\;\mathrm{of}\;\mathrm{device}\\ i_{\left(j,l\right)}^{\left(k\right)}, & \;\mathrm{presence}\;\mathrm{of}\;\mathrm{device} \end{array}\right.,$$

where the index *k* represents the *k*-th device.

## 3. Device Detection

For a desired frame *j* and the *l*-th multipath, the hypothesis containing the information about the user and the device can be represented as

**Hypothesis** **1** **(H1).** *$y_{\left(j,l\right)}=s_{\left(j,l\right)}+n_{\left(j,l\right)},\;$ absence of a device*

**Hypothesis** **2** **(H2).** *$y_{\left(j,l\right)}=s_{\left(j,l\right)}+i_{\left(j,l\right)}+n_{\left(j,l\right)},\;$ presence of a device*

As the main objective is to determine whether another device is present or not, $s_{\left(j,l\right)}$ does not convey any relevant information and it can be subtracted. Thus, yielding the following modified hypothesis

**Hypothesis** **3** **(H3).** *$r_{\left(j,l\right)}=n_{\left(j,l\right)},\;$ absence of a device*

**Hypothesis** **4** **(H4).** *$r_{\left(j,l\right)}=i_{\left(j,l\right)}+n_{\left(j,l\right)},\;$ presence of a device,*

where $r_{\left(j,l\right)}=y_{\left(j,l\right)}-s_{\left(j,l\right)}$. Now, with the modified hypothesis we can easily determine the probability density function (pdf). The pdf of $r_{\left(j,l\right)}$ given $H_{1}$ can be obtained as

(9) $$\begin{array}{c} p\left(r_{\left(j,l\right)};H_{1}\right)=\frac{1}{\sqrt{2\pi }\sigma }\exp\left(-\frac{{\left(r_{\left(j,l\right)}\right)}^{2}}{2\sigma^{2}}\right). \end{array}$$

While, the pdf of $r_{\left(j,l\right)}$ given $H_{2}$ is obtained as

(10) $$\begin{array}{c} p\left(r_{\left(j,l\right)};H_{2}\right)=\frac{1}{\sqrt{2\pi }\sigma }\exp\left(-\frac{{\left(r_{\left(j,l\right)}-i_{\left(j,l\right)}\right)}^{2}}{2\sigma^{2}}\right). \end{array}$$

A likelihood ratio test, which is the ratio between the above two hypotheses yields

(11) $$\begin{array}{ccc} \gamma_{\left(j,l\right)}=\frac{p\left(r_{\left(j,l\right)};H_{2}\right)}{p\left(r_{\left(j,l\right)};H_{1}\right)} & = & \frac{\exp\left(-\frac{{\left(r_{\left(j,l\right)}-i_{\left(j,l\right)}\right)}^{2}}{2\sigma^{2}}\right)}{\exp\left(-\frac{{\left(r_{\left(j,l\right)}\right)}^{2}}{2\sigma^{2}}\right)}\\ & = & \exp\left(\frac{2\left(r_{\left(j,l\right)}\right)\left(i_{\left(j,l\right)}\right)-{\left(i_{\left(j,l\right)}\right)}^{2}}{2\sigma^{2}}\right) \end{array}$$

As $i_{\left(j,l\right)}$ is unknown, we need to replace it with an estimate. In the upcoming section, we will employ characteristic function (CF) to determine the moments of $i_{\left(j,l\right)}$. As the device can enter the network at any moment of time, it is assumed to be uniformly distributed between the transmission of the frame. Furthermore, the impact will have an effect on *K* slots of transmission; therefore, a window-based approach is required which measures this effect. Assuming that the user is impacted over the duration of frame $T_{f}$, and delay $\tau_{m}$, then

(12) $$\begin{array}{c} \Delta \left(m\right)=\frac{1}{T_{f}}\sum_{m=\tau_{m}-T_{f}/2}^{m=\tau_{m}+T_{f}/2}\gamma \left(m\right), \end{array}$$

where m=(jL+l). The effective threshold depending on length *K* will be given as

(13) $$\begin{array}{c} \gamma =\frac{1}{K}\sum_{k=0}^{K}\gamma_{\left(k\right)}. \end{array}$$

Finally the presence of the device is determined as

(14) $$\begin{array}{ccc} \Delta (m) & \leq & \gamma ,\;\mathrm{absence}\mathrm{of}a\mathrm{device}\\ \Delta (m) & > & \gamma ,\;\mathrm{presence}\mathrm{of}a\mathrm{device}. \end{array}$$

Next, the moments of $i_{\left(j,l\right)}$ are derived, which will be used for the device detection.

## 4. Device Presence Modelling

In this section, the mean and variance are calculated when the device is present. This will later be used for designing the Neyman–Pearson criteria. First, the characteristic function (CF) is determined, followed by its moments. With known moments, the mean and variance can be easily calculated. Furthermore, the CF can help measure higher order moments which will help in determining the kurtosis, which can be used for determining the shape of the density as mentioned in [57].

### 4.1. Characteristic Function (CF)

The CF for known *j*-th frame, *l*-th channel tap, conditioned on $\alpha_{k}$ is given as

(15) $$\begin{array}{ccc} \Phi_{i_{\left(j,l\right)}^{\left(k\right)}}\left(\omega \right) & = & \int_{\alpha_{k}}\Phi_{i_{\left(j,l\right)}|\alpha_{k}}\left(\omega \right)f_{\alpha_{k}}\left(\alpha_{k}\right)d\alpha_{k}\\ & = & \frac{1}{T_{f}}\int_{-\frac{T_{f}}{2}}^{\frac{T_{f}}{2}}\exp\left(j\omega h_{\left(l,k\right)}R\left(\alpha_{k}\right)\right)d\alpha_{k}, \end{array}$$

where $\alpha_{k}$ is the time shift between the time hopping code and the *k*-th user, $T_{f}$ is the frame duration and $h_{\left(l,k\right)}$ is the UWB channel characteristics which the device experiences. The device moments can be calculated by the CF of $i_{\left(j,l\right)}^{\left(k\right)}$, which can help in determining the detection and false alarm probability for the UWB system.

### 4.2. Device Presence Moments

The *m*th moment of random variable *X*, with the help of its CF can be evaluated as [58]

(16) $$\begin{array}{c} E\left(X^{m}\right)={\left.{\left(-j\right)}^{m}\frac{d^{m}\Phi_{x}\left(\omega \right)}{d\omega^{m}}\right|}_{\omega =0}. \end{array}$$

Using the above formula, the mean when the user is present is given as

(17) $$\begin{array}{ccc} \mu_{i} & = & E\left((i_{\left(j,l\right)}^{\left(k\right)})\right)\\ & = & {\left.\frac{-j}{T_{f}}\int_{-\frac{T_{f}}{2}}^{\frac{T_{f}}{2}}jh_{\left(l,k\right)}R\left(\alpha_{k}\right)\exp\left(j\omega h_{\left(l,k\right)}R\left(\alpha_{k}\right)\right)d\alpha_{k}\right|}_{\omega =0}\\ & = & \frac{h_{\left(l,k\right)}}{T_{f}}\int_{-\frac{T_{f}}{2}}^{\frac{T_{f}}{2}}R\left(\alpha_{k}\right)d\alpha_{k} \end{array}$$

and the second moment is represented as

(18) $$\begin{array}{ccc} E{\left((i_{\left(j,l\right)}^{\left(k\right)})\right)}^{2}\;\; & = & \;\;{\left.\frac{-1}{T_{f}}\int_{-\frac{T_{f}}{2}}^{\frac{T_{f}}{2}}j^{2}{\left(h_{\left(l,k\right)}\right)}^{2}R^{2}\left(\alpha_{k}\right)\exp\left(j\omega h_{\left(l,k\right)}R\left(\alpha_{k}\right)\right)d\alpha_{k}\right|}_{\omega =0}\\ & = & \frac{{\left(h_{\left(l,k\right)}\right)}^{2}}{T_{f}}\int_{-\frac{T_{f}}{2}}^{\frac{T_{f}}{2}}R^{2}\left(\alpha_{k}\right)d\alpha_{k}\\ & = & {\left(h_{\left(l,k\right)}\right)}^{2}\sigma_{i}^{2}, \end{array}$$

where

(19) $$\begin{array}{ccc} \sigma_{i}^{2} & = & \frac{1}{T_{f}}\int_{-\infty }^{\infty }{\left[\int_{-\infty }^{\infty }\psi_{rec}\left(t-x\right)\psi_{rec}^{*}\left(t\right)dt\right]}^{2}dx\\ & = & \frac{1}{T_{f}}\int_{-\infty }^{\infty }R^{2}\left(x\right)dx. \end{array}$$

Finally, the variance of the presence of user is given as

(20) $$\begin{array}{ccc} \mathrm{Var}\left(i_{\left(j,l\right)}^{\left(k\right)}\right) & = & {\left(h_{\left(l,k\right)}\right)}^{2}\sigma_{i}^{2}-{\left(\frac{h_{\left(l,k\right)}}{T_{f}}\int_{-\frac{T_{f}}{2}}^{\frac{T_{f}}{2}}R\left(\alpha_{k}\right)d\alpha_{k}\right)}^{2}\\ & = & {\left(h_{\left(l,k\right)}\right)}^{2}\sigma_{i}^{2}-\frac{{\left(h_{\left(l,k\right)}\right)}^{2}\sigma_{i}^{2}}{T_{f}}\\ & = & {\left(h_{\left(l,k\right)}\right)}^{2}\sigma_{i}^{2}\left(1-\frac{1}{T_{f}}\right) \end{array}$$

## 5. Performance Analysis

In this section, the probability of false alarm $P_{FA}$ and probability of detection $P_{D}$ are calculated.

### 5.1. Probability of False Alarm

The probability of false alarm $P_{FA}$ is given as

(21) $$\begin{array}{ccc} P_{FA} & = & P\{\Delta (m)>\gamma |\mathrm{no}\mathrm{device}\mathrm{is}\mathrm{present}\}\\ & = & \int_{\gamma }^{\infty }\frac{1}{\sqrt{2\pi }\sigma_{a}}\exp\left(-\frac{{\left(x-\mu_{a}\right)}^{2}}{2\sigma_{a}^{2}}\right)dx\\ & = & Q\left(\frac{\gamma -\mu_{a}}{\sigma_{a}}\right), \end{array}$$

where Q(·) is the standard Gaussian Q-function, given as

(22) $$\begin{array}{c} \;Q\left(t\right)=\frac{1}{\sqrt{2\pi }}\int_{t}^{\infty }\exp\left(-\frac{t^{2}}{2}\right)dt. \end{array}$$

and $\mu_{a}$ and $\sigma_{a}^{2}$ are calculated in (A2) and (A7), respectively, as shown in Appendix A.

### 5.2. Probability of Detection

The probability of detection $P_{D}$ is given as

(23) $$\begin{array}{ccc} P_{D} & = & P\{\Delta (m)>\gamma |\mathrm{device}\mathrm{is}\mathrm{present}\}\\ & = & \int_{\gamma }^{\infty }\frac{1}{\sqrt{2\pi }\sigma_{p}}\exp\left(-\frac{{\left(x-\mu_{p}\right)}^{2}}{2\sigma_{p}^{2}}\right)dx\\ & = & Q\left(\frac{\gamma -\mu_{p}}{\sigma_{p}}\right), \end{array}$$

where $\mu_{p}$ and $\sigma_{p}^{2}$ are calculated in (A9) and (A14), respectively, as shown in Appendix B.

### 5.3. Optimized Threshold γ

In this section, we try to relate the optimized threshold γ. For $P_{FA}$, the *Q*-function is monotonically decreasing for $\frac{\gamma -\mu_{a}}{\sigma_{a}}\geq 0$. Therefore, from (21), the threshold γ is calculated as

(24) $$\begin{array}{c} \gamma =\mu_{a}+\sigma_{a}Q^{-1}\left(P_{FA}\right) \end{array}$$

Substituting the above in (23), we get

(25) $$\begin{array}{ccc} P_{D} & = & Q\left(\frac{\mu_{a}+\sigma_{a}Q^{-1}\left(P_{FA}\right)-\mu_{p}}{\sigma_{p}}\right) \end{array}$$

## 6. Performance Analysis and Discussion

In this section, the performance of the receiver is discussed by using the probability of false alarm and detection as calculated in the previous section. We will first look at the affect of the pulse duration on the probability of false alarm followed by the effects of interference noise ratio (INR) and transmitted energy of the interference on the UWB system. Usually, the change in environment due to the presence of the device is modelled as a sinusoidal [47,50,51,59,60]. The two unknown parameters, frequency and time shift, help in determining the presence of a device. Furthermore, the device will be moving indoors and the speed will be less than 5 km/h, which will result in a normalized doppler frequency of $1\times 10^{-7}$, having a very slow effect on the channel coefficients. The simulations are performed based on the IEEE 802.15.4a channel model final report [54]. However, the SV parameters were simulated using an indoor industrial environment [53], as it is corresponds to the most challenging environment with a large number of reflections. Note that, our simulations were carried out based on the Saleh–Valenzuela (S–V) channel model, which is characterized by the parameters 1/Λ=14.11 ns, cluster decay rate =2.63 ns and ray power decay =4.58 ns. The term INR is defined as $INR=\sigma_{i}^{2}/\sigma^{2}$. The simulation parameters are shown in Table 1.

**Figure 1:**
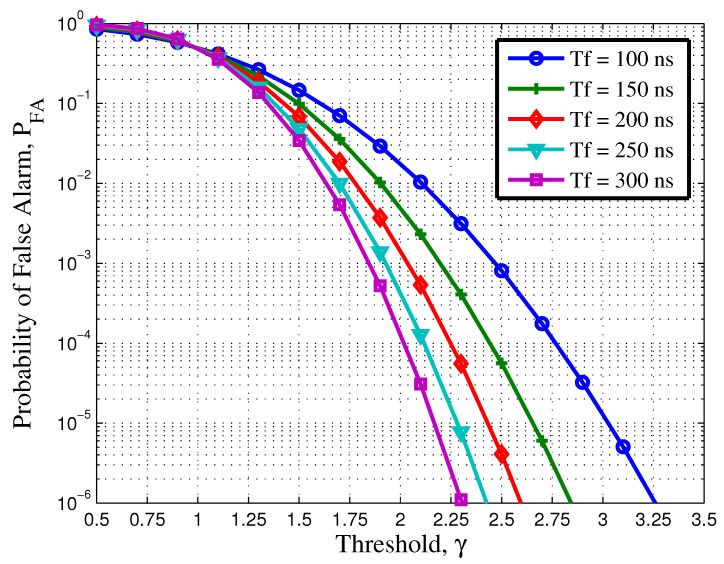
Probability of false alarm versus threshold when using different frame duration at interference noise ratio (INR) =5 dB.

Let us study the effects of threshold over the frame duration. Figure 1 shows the false alarm probability as a function of the threshold γ for different values of frame duration. These curves are plotted with the help of (23), where $\mu_{a}$ and $\sigma_{a}$ are calculated using (29) and (34), respectively. In this simulation, the INR was fixed to 5 dB. It can be observed that as the frame duration increases, the value of threshold decreases. For a false alarm probability equal to $10^{-6}$, it can be observed that a threshold of 2.26 will be required to detect a frame length of 300 ns. However, for a frame length of 100 ns, the threshold value should be around 3.25. This satisfies the result as calculated in (A7). From (A7), it can be analysed that as the frame duration $T_{f}$ increases, $\sigma_{a}^{2}$ decreases, resulting in a lower value of γ, as shown in (24). It can be also observed from the figure that for $\gamma =\mu_{a}=1$, the $P_{FA}=0.5$.

**Figure 2:**
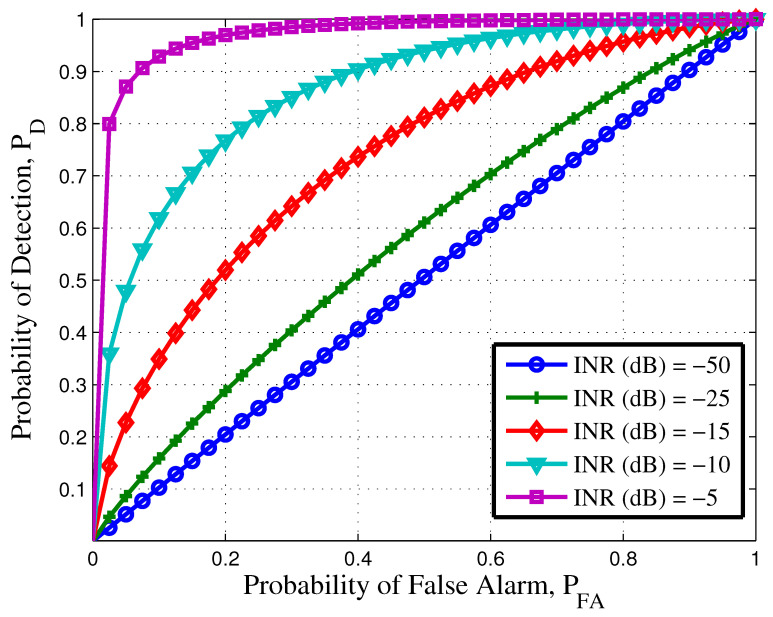
Receiver operating characteristics for UWB system with different interference to noise ratio (INR).

In Figure 2, NP criteria is adopted to calculate the detection probability ($P_{D}$) and false alarm probability ($P_{FA}$) in STH-UWB systems. Figure 2 was simulated using (23) and (27). The value for $\mu_{a}$, $\mu_{p}$, $\sigma_{a}$ and $\sigma_{p}$ are calculated using (29), (36), (34) and (41), respectively. Figure 2 illustrates the effect of interference to noise ratio (INR). From this figure, it is obvious that as the INR increases the $P_{D}$ improves and $P_{FA}$ decreases, which results in easier detection of the device. It can be observed from Figure 2 that, for $P_{FA}=0.2$, the device is detected with probability 0.98, when an INR of −5 dB. However, as the INR decreases $P_{D}$ will be reduced significantly to 80% and below depending upon the INR value. With these receiver operating characteristics (ROC)s for STH-UWB radar can be designed. This threshold detection will help increase the $P_{D}$ and reduce $P_{FA}$ for a given INR.

**Figure 3:**
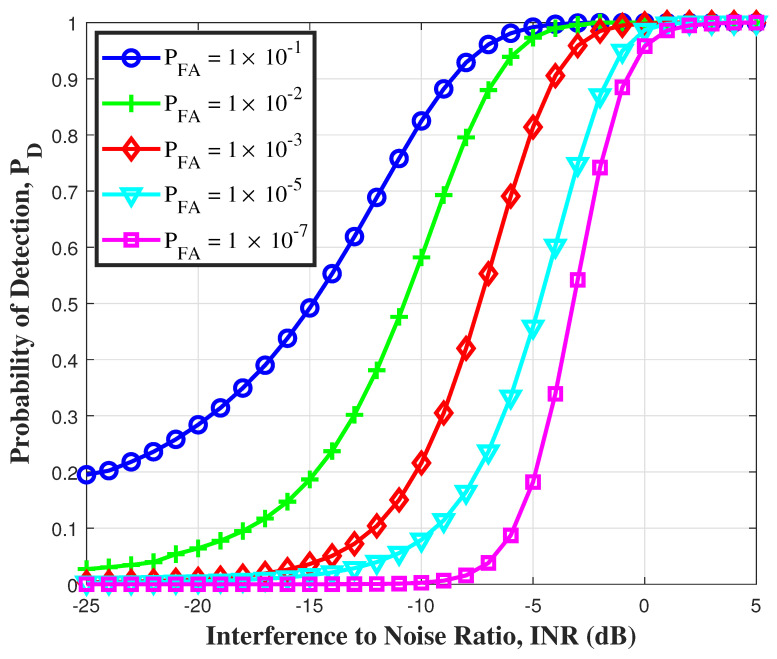
Detection performance of UWB system on linear $P_{FA}$ scale.

In Figure 3, it is observed that for a given $P_{FA}$, the detection performance increases monotonically with the INR. The pulse duration was fixed to $T_{f}=100$ ns. Figure 3 was simulated using (27), where the value for $\mu_{p}$ and $\sigma_{p}$ are calculated using (36) and (41), respectively. From the figure, it becomes obvious that in order to improve the detection performance, either $P_{FA}$ is increased or the INR is increased. For a fixed $P_{FA}=1\times 10^{-5}$, we need an improvement of 3 dB in INR to improve the detection probability from 0.3 to 0.8. However, for lower $P_{FA}=0.1$, we will require an improvement of 8 dB to reach the same detection probability. Therefore, it can be concluded from the figure that for higher $P_{FA}$, higher increase in INR is required as compared to lower $P_{FA}$.

**Figure 4:**
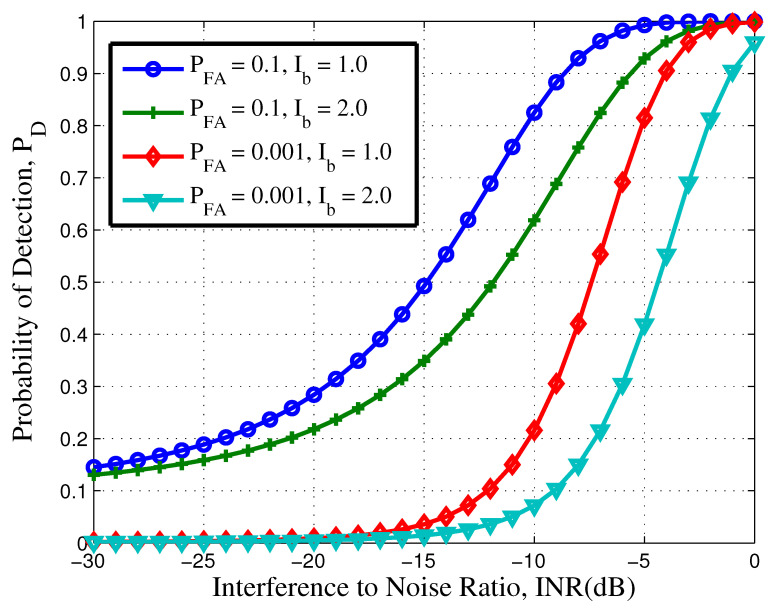
Detection performance and effects of interference energy.

In order to further clarify this concept, we have plotted Figure 4. Figure 4 shows the effect of increasing the energy of the interference. The pulse duration was fixed to $T_{f}=100$ ns. Figure 4 was simulated using (27), where the value for $\mu_{p}$ and $\sigma_{p}$ are calculated using (36) and (41), respectively. By doubling the interference energy, there is a improvement of 3 dB in INR for a fixed $P_{FA}=0.1$ and $P_{D}=0.5$. The other main point of Figure 4 is found when comparing the performance of IR UWB system to DS-UWB system. In IR-UWB, there is only a single transmitted pulse in a frame duration, while for a DS system, $N_{s}$ pulses are transmitted in each frame, resulting in a decrease of transmitted power. If a DS-UWB system transmits two pulses in a frame, then there will be a decrease of 3 dB in INR for a fixed $P_{FA}$ at $P_{D}=0.5$.

**Figure 5:**
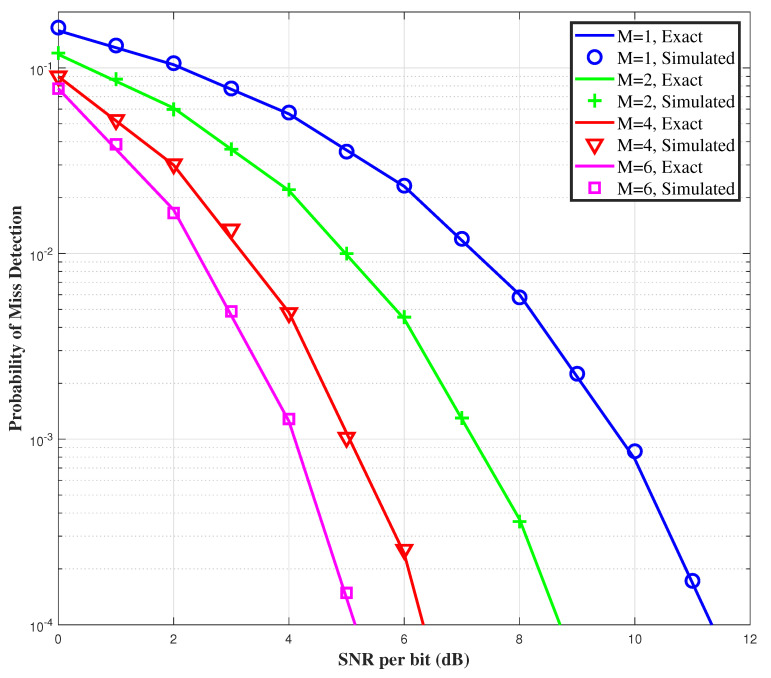
Simulated and calculated Probability of miss detection curve comparison for STH M−ary PPM System. The curves labelled as “Exact” are plotted using (27) where as the markers denote the simulation performance using (16).

The performance of the proposed detection method is corroborated with the derived analytical expressions of probability of miss-detection. The performance of the simulated algorithm matches exactly with the derived theoretical performance expressions, which indicates that the algorithm achieves benchmark performance. Moreover, the derived expressions of $P_{FA}$ and $P_{D}$ can be utilized to plot the ROC and determine the operating point for the algorithm in various environments. Figure 5 corroborates the derived analytical expression of the probability of miss-detection, i.e., $P_{MD}=1-P_{D}$, through simulation. The probability of miss-detection is plotted with varying the SNR per bit and the parameter *M*. A general trend observed is that the miss-detection probability reduces as the SNR increases. In addition, it can be observed that as *M* increases, the miss-detection probability reduces. This trend is observed because *M* indicates that the transmission time is divided into *M* orthogonal slots. Each user randomly chooses a slot for transmission and this reduces the collision/interference probability. As a result, as *M* increases, the miss-detection probability reduces due to lower interference. Finally, it can be noted that the simulation results match exactly with the derived analytical expression.

## 7. Conclusions

In this paper, a novel device-free detection method is proposed for an indoor STH-PPM UWB system. Our method relies solely on calculating the characteristic function (CF) of the device. 1st and 2nd moments are calculated using this CF, to develop decision statistics which are employed to design and develop the receiver operating characteristics (ROC)s. Particularly, it was shown that $P_{FA}$ can be reduced by increasing the detection threshold and/or frame length. Furthermore, probability of detection approaches 1 for an interference-to-noise ratio greater than −5 dB, even for a very low probability of false alarm. Through numerical simulations, it is shown that the proposed detection method is able to detect the presence of the device and results in satisfactory performance with a reduced probability of false alarm. Moreover, the simulation results corroborated the derived performance expressions. The proposed detector can be implemented in a real time system. As a future work, similar to [17], we are planning to implement and test this algorithm on a UWB kit and carry out the measurements in an industrial warehouse.

## Figures and Tables

**Table 1:** The path loss and small-scale fading parameters for the simulated line of sight (LoS) Industrial ultra-wide bandwidth (UWB) channel, as mentioned in [53,54].

Path Loss exponent	n	1.2
Shadowing Standard Deviation	$\sigma_{s}$	6 dB
Path Loss at 1 m distance	$PL_{0}$	56.7 dB
Antenna Loss	$A_{ant}$	3 dB
Frequency dependence of Path Loss	κ	−1.103
Nakagami-*m* factor mean	$m_{0}$	0.36 dB
Nakagami-*m* factor variance	${\hat{m}}_{0}$	1.13
Nakagami-*m* for strong components	${\tilde{m}}_{0}$	12.99 dB

## Data Availability

Not applicable.

## Abbreviations

The following abbreviations are used in this manuscript:

AWGN	Added White Gaussian Noise
BER	Bit Error Rate
BLE	Bluetooth Low Energy
CF	Characteristic Function
COVID	Coronavirus Disease
CRLB	Cramer-Rao Lower Bounds
DS	Direct-Sequence
DS-TH	Direct-Sequence and Time-Hopping
False Alarm	FA
GPS	Global Positioning System
INR	Interference Noise Ratio
IR	Impulse Radio
LoRa	Long Range
LoS	Line of Sight
NLoS	Non Line of Sight
NP	Neyman Pearson
OOK	On-Off Keying
PAM	Pulse Amplitude Modulation
PPM	Pulse Position Modulation
PSM	Pulse Shape Modulation
RFID	Radio Frequency Identification
ROC	Receiver Operating Characteristics
STH	Slow Time Hopping
SV	Saleh Valenzuela
TDoA	Time Difference of ArrivalChannel State Information
ToA	Time of Arrival
UWB	Ultra-wide bandwidth
WiFi	Wireless Fidelity

## List of Symbols

The following symbols are used in this manuscript:

s(t)	Transmitted Signal
y(t)	Received Signal
n(t)	AWGN Noise
ψ(t)	Time domain UWB pulse
$N_{s}$	Number of pulses
*j*	index of Frame
*t*	index of Time
*k*	index of Device
h(t)	Channel Impulse Response
*V*	Number of Clusters
*U*	Number of Rays in Clusters
$T_{f}$	Frame duration
*L*	Total Number of multipath
$T_{l}$	Delay of the *l*-th multipath
*K*	Number of devices
$h_{l}$	Channel impulse response of *l* multipath
$\sigma^{2}$	AWGN Noise Variance
γ	Threshold
$E(X^{m})$	*m*-th moment
$\mu_{a}$	Mean when Deviceis Absent
$\mu_{p}$	Mean when Device is Present
$\sigma_{a}$	Variance when Device is Absent
$\sigma_{p}$	Variance when Device is Present
R(·)	Autocorrelation of the UWB pulse
δ(·)	Dirac delta Function

## Appendix A

In this appendix, we will calculate the mean and variance when the device is not present. In the absence of the user, the sliding window is given as

(A1) $$\begin{array}{c} \Delta \left(m|\mathrm{no}\mathrm{device}\mathrm{is}\mathrm{present}\right)=\frac{1}{T_{f}}\sum_{m=\tau_{m}-T_{f}/2}^{\tau_{m}+T_{f}/2}\gamma \left(m|H_{1}\right), \end{array}$$

The mean of the above can be calculated as

(A2) $$\begin{array}{ccc} \mu_{a}\;\;\; & = & E\left\{\Delta (m|\mathrm{no}\mathrm{device}\mathrm{is}\mathrm{present})\right\},\\ & = & E\left\{\frac{1}{T_{f}}\sum_{m=\tau_{m}-T_{f}/2}^{\tau_{m}+T_{f}/2}\gamma \left(m|H_{1}\right)\right\},\\ & = & \frac{1}{T_{f}}\sum_{m=\tau_{m}-T_{f}/2}^{\tau_{m}+T_{f}/2}E\left\{\gamma \left(m|H_{1}\right)\right\},\\ & = & \frac{1}{T_{f}}\sum_{m=\tau_{m}-T_{f}/2}^{\tau_{m}+T_{f}/2}\int_{-\infty }^{\infty }\;\;\;\;\exp\left(\frac{2r_{m}i_{m}-i_{m}^{2}}{2\sigma^{2}}\right)\frac{1}{\sqrt{2\pi }\sigma }\exp\left(-\frac{r_{m}^{2}}{2\sigma^{2}}\right)dr_{m},\\ & = & \frac{1}{T_{f}}\sum_{m=\tau_{m}-T_{f}/2}^{\tau_{m}+T_{f}/2}\underset{1}{\underset{︸}{\frac{1}{\sqrt{2\pi }\sigma }\int_{-\infty }^{\infty }\exp\left(-\frac{{\left(r_{m}-i_{m}\right)}^{2}}{2\sigma^{2}}\right)dr_{m}}},\\ & = & \frac{1}{T_{f}}\sum_{m=\tau_{m}-T_{f}/2}^{\tau_{m}+T_{f}/2}1\\ & = & 1. \end{array}$$

Let us now calculate the second moment.

(A3) $$\begin{array}{ccc} & \; & E\{\Delta^{2}\left(m,\tilde{m}|\mathrm{no}\mathrm{device}\mathrm{is}\mathrm{present}\right)\}\\ & = & E\left\{\left(\frac{1}{T_{f}}\sum_{m=\tau_{m}-T_{f}/2}^{\tau_{m}+T_{f}/2}\gamma \left(m|H_{1}\right)\right)\left(\frac{1}{T_{f}}\sum_{\tilde{m}=\tau_{\tilde{m}}-T_{f}/2}^{\tau_{\tilde{m}}+T_{f}/2}\gamma \left(\tilde{m}|H_{1}\right)\right)\right\}\\ & = & \frac{1}{T_{f}^{2}}\sum_{m=\tau_{m}-T_{f}/2}^{\tau_{m}+T_{f}/2}\sum_{\tilde{m}=\tau_{\tilde{m}}-T_{f}/2}^{\tau_{\tilde{m}}+T_{f}/2}E\left\{\gamma \left(m|H_{1}\right)\gamma \left(\tilde{m}|H_{1}\right)\right\} \end{array}$$

When $m\neq \tilde{m}$, the above can be given as

(A4) $$\begin{array}{ccc} E\{\gamma \left(m|H_{1}\right)\gamma \left(\tilde{m|H_{1}}\right)\} & = & E\left\{\gamma \left(m|H_{1}\right)\right\}E\left\{\gamma \left(\tilde{m}|H_{1}\right)\right\}\\ & = & 1. \end{array}$$

While, for $m=\tilde{m}$, the above reduces to

(A5) $$\begin{array}{ccc} & \; & E\left\{\gamma \left(m|H_{1}\right)\gamma \left(m|H_{1}\right)\right\}=E\left\{\gamma {\left(m|H_{1}\right)}^{2}\right\}\\ & = & \int_{-\infty }^{\infty }\exp\left(\frac{2r_{m}i_{m}-i_{m}^{2}}{\sigma^{2}}\right)\frac{1}{\sqrt{2\pi }\sigma }\exp\left(-\frac{r_{m}^{2}}{2\sigma^{2}}\right)dr_{m},\\ & = & \exp\left(\frac{i_{m}^{2}}{\sigma^{2}}\right)\underset{1}{\underset{︸}{\frac{1}{\sqrt{2\pi }\sigma }\int_{-\infty }^{\infty }\exp\left(-\frac{{\left(r_{m}-2i_{m}\right)}^{2}}{2\sigma^{2}}\right)dr_{m}}},\\ & = & \exp\left(\frac{h_{m}^{2}\sigma_{i}^{2}}{\sigma^{2}}\right) \end{array}$$

where $\sigma^{2}$ is the variance of the AWGN and $i_{m}^{2}=h_{m}^{2}\sigma_{i}^{2}$ according to (18). Now, substituting (A4) and (A5) in (A3), we obtain

(A6) $$\begin{array}{ccc} E\{\Delta^{2}\left(m,\tilde{m}|\mathrm{no}\mathrm{device}\mathrm{is}\mathrm{present}\right)\} & = & \frac{1}{T_{f}^{2}}\left(T_{f}\left(\exp\left(\frac{h_{m}^{2}\sigma_{i}^{2}}{\sigma^{2}}\right)\right)+\left(T_{f}^{2}-T_{f}\right)1\right)\\ & = & 1+\frac{1}{T_{f}}\left(\exp\left(\frac{h_{m}^{2}\sigma_{i}^{2}}{\sigma^{2}}\right)-1\right) \end{array}$$

Finally, the variance is given as

(A7) $$\begin{array}{ccc} \sigma_{a}^{2} & = & E\left\{\Delta^{2}\left(m,\tilde{m}|\mathrm{no}\mathrm{device}\mathrm{is}\mathrm{present}\right)\right\}-E{\left\{\Delta \left(m|\mathrm{no}\mathrm{device}\mathrm{is}\mathrm{present}\right)\right\}}^{2}\\ & = & 1+\frac{1}{T_{f}}\left(\exp\left(\frac{h_{m}^{2}\sigma_{i}^{2}}{\sigma^{2}}\right)-1\right)-1\\ & = & \frac{1}{T_{f}}\left(\exp\left(\frac{h_{m}^{2}\sigma_{i}^{2}}{\sigma^{2}}\right)-1\right) \end{array}$$

## Appendix B

In this appendix, we will calculate the mean and variance when the device is present. In the presence of the device, the sliding window is given as

(A8) $$\begin{array}{c} \Delta \left(m|\mathrm{device}\mathrm{is}\mathrm{present}\right)=\frac{1}{T_{f}}\sum_{m=\tau_{m}-T_{f}/2}^{\tau_{m}+T_{f}/2}\gamma \left(m|H_{2}\right). \end{array}$$

The mean of the above can be calculated as

(A9) $$\begin{array}{ccc} \mu_{p} & = & E\left\{\Delta \left(m|\mathrm{device}\mathrm{is}\mathrm{present}\right)\right\}=E\left\{\frac{1}{T_{f}}\sum_{m=\tau_{m}-T_{f}/2}^{\tau_{m}+T_{f}/2}\gamma \left(m|H_{2}\right)\right\}\\ & = & \frac{1}{T_{f}}\sum_{m=\tau_{m}-T_{f}/2}^{\tau_{m}+T_{f}/2}E\left\{\gamma \left(m|H_{2}\right)\right\}\\ & = & \frac{1}{T_{f}}\sum_{m=\tau_{m}-T_{f}/2}^{\tau_{m}+T_{f}/2}E\left\{\exp\left(\frac{2r_{m}i_{m}-i_{m}^{2}}{2\sigma^{2}}\right)\right\}\\ & = & \;\;\;\;\frac{1}{T_{f}}\;\;\sum_{m=\tau_{m}-T_{f}/2}^{\tau_{m}+T_{f}/2}\;\int_{-\infty }^{\infty }\;\;\;\;\exp\left(\frac{2r_{m}i_{m}-i_{m}^{2}}{2\sigma^{2}}\right)\;\;\frac{1}{\sqrt{2\pi }\sigma }\exp\left(-\frac{{\left(r_{m}-i_{m}\right)}^{2}}{2\sigma^{2}}\right)dr_{m}\\ & = & \frac{1}{T_{f}}\sum_{m=\tau_{m}-T_{f}/2}^{\tau_{m}+T_{f}/2}\exp\left(\frac{i_{m}^{2}}{\sigma^{2}}\right)\underset{1}{\underset{︸}{\int_{-\infty }^{\infty }\frac{1}{\sqrt{2\pi }\sigma }\exp\left(-\frac{{\left(r_{m}-2i_{m}\right)}^{2}}{2\sigma^{2}}\right)dr_{m}}}\\ & = & \frac{1}{T_{f}}\sum_{m=\tau_{m}-T_{f}/2}^{\tau_{m}+T_{f}/2}\exp\left(\frac{h_{m}^{2}\sigma_{i}^{2}}{\sigma^{2}}\right) \end{array}$$

The second moment is calculated as

(A10) $$\begin{array}{ccc} & \; & E\{\Delta^{2}\left(m,\tilde{m}|\mathrm{device}\mathrm{is}\mathrm{present}\right)\}\\ & = & E\left\{\left(\frac{1}{T_{f}}\sum_{m=\tau_{m}-T_{f}/2}^{\tau_{m}+T_{f}/2}\gamma \left(m|H_{2}\right)\right)\left(\frac{1}{T_{f}}\sum_{\tilde{m}=\tau_{\tilde{m}}-T_{f}/2}^{\tau_{\tilde{m}}+T_{f}/2}\gamma \left(\tilde{m}|H_{2}\right)\right)\right\}\\ & = & \frac{1}{T_{f}^{2}}\sum_{m=\tau_{m}-T_{f}/2}^{\tau_{m}+T_{f}/2}\sum_{\tilde{m}=\tau_{\tilde{m}}-T_{f}/2}^{\tau_{\tilde{m}}+T_{f}/2}E\left\{\gamma \left(m|H_{2}\right)\gamma \left(\tilde{m}|H_{2}\right)\right\} \end{array}$$

When $m\neq \tilde{m}$, the above can be given as

(A11) $$\begin{array}{ccc} E\{\gamma \left(m|H_{2}\right)\gamma \left(\tilde{m}|H_{2}\right)\} & = & E\left\{\gamma \left(m|H_{2}\right)\right\}E\left\{\gamma \left(\tilde{m}|H_{2}\right)\right\}\\ & = & \exp\left(\frac{h_{m}^{2}\sigma_{i}^{2}}{\sigma^{2}}\right)\exp\left(\frac{h_{\tilde{m}}^{2}\sigma_{i}^{2}}{\sigma^{2}}\right)\\ & = & \exp\left(\frac{\left(h_{m}^{2}+h_{\tilde{m}}^{2}\right)\sigma_{i}^{2}}{\sigma^{2}}\right) \end{array}$$

While for $m=\tilde{m}$, the above reduces to

(A12) $$\begin{array}{ccc} & \; & E\{\gamma \left(m|H_{2}\right)\gamma \left(m|H_{2}\right)\}\\ & = & E\{\gamma {\left(m|H_{2}\right)}^{2}\}\\ & = & \int_{-\infty }^{\infty }{\left(\exp\left(\frac{2r_{m}i_{m}-i_{m}^{2}}{2\sigma^{2}}\right)\right)}^{2}\frac{1}{\sqrt{2\pi }\sigma }\exp\left(-\frac{{\left(r_{m}-i_{m}\right)}^{2}}{2\sigma^{2}}\right)dr_{m}\\ & = & \exp\left(\frac{3i_{m}^{2}}{\sigma^{2}}\right)\underset{1}{\underset{︸}{\frac{1}{\sqrt{2\pi }\sigma }\int_{-\infty }^{\infty }\exp\left(-\frac{{\left(r_{m}-3i_{m}\right)}^{2}}{2\sigma^{2}}\right)dr_{m}}}\\ & = & \exp\left(\frac{3h_{m}^{2}\sigma_{i}^{2}}{\sigma^{2}}\right) \end{array}$$

Now, substituting (A11) and (A12) in (A10), we get

(A13) $$\begin{array}{ccc} & \; & E\{\Delta^{2}\left(m,\tilde{m}|\mathrm{device}\mathrm{is}\mathrm{present}\right)\}\\ & = & \frac{1}{T_{f}^{2}}\left(T_{f}\exp\left(\frac{3h_{m}^{2}\sigma_{i}^{2}}{\sigma^{2}}\right)+\left(T_{f}^{2}-T_{f}\right)\exp\left(\frac{\left(h_{m}^{2}+h_{\tilde{m}}^{2}\right)\sigma_{i}^{2}}{\sigma^{2}}\right)\right)\\ \;\;\;\;\; & = & \;\;\;\;\exp\left(\;\frac{\left(h_{m}^{2}+h_{\tilde{m}}^{2}\right)\sigma_{i}^{2}}{\sigma^{2}}\right)\;\;\;+\;\frac{1}{T_{f}}\;\left(\;\exp\;\left(\frac{3h_{m}^{2}\sigma_{i}^{2}}{\sigma^{2}}\right)\;-\;\;\exp\;\left(\frac{\left(h_{m}^{2}+h_{\tilde{m}}^{2}\right)\sigma_{i}^{2}}{\sigma^{2}}\right)\;\right) \end{array}$$

Finally, the variance will be given as

(A14) $$\begin{array}{ccc} \sigma_{p}^{2} & = & E\left\{\Delta^{2}\left(m,\tilde{m}|\mathrm{device}\mathrm{is}\mathrm{present}\right)\right\}-E{\left\{\Delta \left(m|\mathrm{device}\mathrm{is}\mathrm{present}\right)\right\}}^{2}\\ & = & \exp\left(\;\frac{\left(h_{m}^{2}\;+\;h_{\tilde{m}}^{2}\right)\sigma_{i}^{2}}{\sigma^{2}}\right)\;\;\;+\;\frac{1}{T_{f}}\;\left(\;\exp\;\left(\frac{3h_{m}^{2}\sigma_{i}^{2}}{\sigma^{2}}\right)\;-\;\;\exp\;\left(\frac{\left(h_{m}^{2}\;+\;h_{\tilde{m}}^{2}\right)\sigma_{i}^{2}}{\sigma^{2}}\right)\;\right)\\ & \;-\; & \exp\left(\;\frac{\left(h_{m}^{2}+h_{\tilde{m}}^{2}\right)\sigma_{i}^{2}}{\sigma^{2}}\right)\;\;\;-\;\frac{1}{T_{f}}\;\left(\;\exp\;\left(\frac{2h_{m}^{2}\sigma_{i}^{2}}{\sigma^{2}}\right)\;-\;\;\exp\;\left(\frac{\left(h_{m}^{2}+h_{\tilde{m}}^{2}\right)\sigma_{i}^{2}}{\sigma^{2}}\right)\;\right)\\ & = & \frac{1}{T_{f}}\left(\exp\left(\frac{3h_{m}^{2}\sigma_{i}^{2}}{\sigma^{2}}\right)\;-\;\exp\left(\frac{2h_{m}^{2}\sigma_{i}^{2}}{\sigma^{2}}\right)\right) \end{array}$$
